# Impact of COVID-19 on Portuguese Dental Students: A Cohort Study

**DOI:** 10.3390/healthcare11060818

**Published:** 2023-03-10

**Authors:** Rodrigo Melo dos Santos Gonçalves, Gustavo Vicentis Oliveira Fernandes, Juliana Campos Hasse Fernandes, Mariana Seabra, Andreia Figueiredo

**Affiliations:** 1 Faculty of Dental Medicine, Universidade Católica Portuguesa, 3504-505 Viseu, Portugal; 2 Periodontics and Oral Medicine Department, University of Michigan School of Dentistry, Ann Arbor, MI 48109, USA; 3 Center for Interdisciplinary Research in Health (CIIS), Universidade Católica Portuguesa, 3504-505 Viseu, Portugal; 4 Independent Researcher, Ann Arbor, MI 48109, USA

**Keywords:** COVID-19, temporomandibular disorders, anxiety, depression, academic performance, dentistry, pandemics

## Abstract

Introduction: The goal of this study was to assess the impact of COVID-19 on Portuguese dental students on their depression, anxiety, temporomandibular dysfunction, academic degree, and oral behaviors. Methods: The target of this population study was to enroll third-, fourth-, and fifth-year students of the Integrated Master’s degree in Dental Medicine at the Universidade Católica Portuguesa—Faculty of Dental Medicine (Viseu, Portugal) in the academic year 2021–2022. Temporomandibular disorders (TMD) symptoms, oral behaviors, anxiety, and depression were assessed using validated questionnaires. The variables under study were (a) age, gender, marital status, academic level, academic degree, medication, and any existing pathologies; (b) questions related to taste changes or thoughts that would indicate (or not) worsening psychological conditions (anxiety and depression). After data collection (Google Forms^®^), data were transferred to an Excel file and entered into SPSS^®^ software. A chi-square test allowed the independence between ordinal or nominal variables. The Spearman correlation test was used to analyze the relationship between variables in the study (significant if *p* ≤ 0.05). Results: 98.2% of the students answered the questionnaire. TMD had a prevalence of 35%, and anxiety and depression a prevalence of 29% and 24%, respectively. The results showed that the female gender was the most affected by psychological and temporomandibular disorders. Statistically significant correlations were identified between variables, such as gender and anxiety, TDM, and depression (respectively, *p* = 0.0001, *p* = 0.014, and *p* = 0.026); between TDM and anxiety and depression (respectively, *p* = 0.001 and *p* < 0.001); and between performing oral behaviors and TMD, anxiety, and depression (respectively, *p* = 0.0001, *p* = 0.0001, and *p* = 0.006). The variables, such as age and academic degree, did not establish a statistically significant relationship with TMD, oral behaviors, and the two psychological conditions. Conclusions: It was possible to conclude that there was a moderate prevalence of TMD, anxiety, and depression in the period studied. Moreover, a statistically significant relationship was found between TMD, anxiety, and depression with gender and frequency of OBs; however, no significant association was found for TMD, anxiety, and depression with the age of students and with the academic degree.

## 1. Introduction

The emergence of the SARS-CoV-2 virus led to many studies worldwide to mitigate the disease’s effects [1,2]. The WHO declared COVID-19 [3] a Public Health Emergency of International Concern (PHEIC) due to the virus’s rapid spread. This decision aimed to improve coordination, cooperation, and global solidarity to prevent the spread of the virus [4,5,6]. The infected patients had characteristic symptoms, such as fever, cough, rhinitis, fatigue, and shortness of breath [3,7]. Current studies show an enhanced understanding of COVID-19′s etiology and effects as they pursue the development of effective countermeasures for its treatment [3]. Because of the disease’s rapid evolution, acting swiftly and activating contingency plans was necessary [4,8]. The European Union approved several measures to avoid the disease spreading [9]. Nevertheless, the subsequent confinement forced four billion citizens to be restricted to their homes and prohibited from socializing, leaving psychological sequelae [10]. The pandemic created and developed climates of uncertainty, stress, and anxiety that harmed people’s mental health [10,11,12,13,14,15,16] in ways that cannot be overcome without medical support [17]. Moreover, an infected person can experience great psychological pressure, with social isolation, which can cause higher levels of anxiety and depression [11,18].

Anxiety is one of the most common mental disorders [19] and is associated with depressive disorder. Both anxiety and depression constitute a problem that has spurred several studies and investigations, whether on the causes or the consequences of various pathologies, such as the high number of suicides [20]. In fact, in 2018, Portugal had the second highest rate of psychological disorders in Europe due to its high rate of anxiety [21,22]. The global study of depression and anxiety disorders’ prevalence and burden (in 204 countries and territories) in 2020 due to the COVID-19 pandemic concluded that there was a 28% increase in cases of depression (53 million people) and a 26% increase in cases of anxiety disorders (73 million people), with young people and women being the most affected groups in terms of mental health [23].

Depression and anxiety may be associated with the etiology of temporomandibular disorders (TMDs) [24]. TMDs have a multifactorial etiology and are defined as a condition that involves alterations in the structures and/or function of the stomatognathic system in relation to the temporomandibular joint and its musculoskeletal structures [25,26]. In addition, its etiology is also affected by several factors, such as environmental, biological, psychological, biomechanical, and/or neuromuscular factors [24,25,26,27]. These disorders are often considered a subclass of musculoskeletal disorders, causing pain in the orofacial region, and are included in the diagnostic criteria for TMD [28]. The treatment option for TMD comes from the correct diagnosis of its etiology and classification regarding the stage of evolution [29]. Clinical evidence alludes to the correlation between TMD and psychological problems, depression, and anxiety [24]. This correlation has achieved greater relevance in our society during and after the pandemic period, with the possibility of drawing these conclusions due to the significant increase in demand for mental health professionals after the end of confinement, as well as the collection of data or questionnaires applied to society that confirm this association [25,26,27,28,29]. However, it is also possible to find studies in the literature that contradict the possible existence of an association between mental health problems and the pandemic [26,30,31,32].

Literature on the impact of SARS-CoV-2 infection on patients with anxiety and depression is still sparse. Recent studies confirm that vaccinated and unvaccinated healthcare staff have experienced anxiety due to the pandemic, which can be reduced through immunization, resulting in lower stress [33]. Another study showed that students must have the right mindset to understand that many changes will exist until COVID-19 is under control and the lockdown period is over [34].

Thus, the aim of the present research, to the best of our knowledge and reported for the first time in the literature (Portuguese dental students and involving oral and systemic questions), was to describe and compare the possible implications of compulsory confinement periods related to COVID-19 on the health of Dental Medicine students in order to verify their oral, physiological, and psychological behavior. Specifically, the study aimed to (i) verify the type of relationship among the variables studied, and (ii) analyze the prevalence of the above problems in the students.

## 2. Material and Methods

This study was approved by the Ethics Committee on Human Research of the UCP (number 194, Portugal) and followed the Declaration of Helsinki (1975, updated 2013). All students received information about the nature of the study and agreed to authorize the publication, reproduction, and dissemination of the paper in public access areas on the internet. Data processing was permitted by themselves. Personal data was made anonymous. The Faculty’s Data Protection Officer (DPO) was referred to in the informed consent. There was no conflict of interest. Neither were there any financial or personal relationships that may have been inappropriately biased or influenced the results. Moreover, FMD was made available to the participants on the evidence of the impact of the COVID-19 pandemic, eventually supporting them with any necessary aspect.

### 2.1. Population and Sample

The study’s target population consisted of students in their third, fourth, and fifth years of the Integrated Master of Dental Medicine, Faculty of Dental Medicine of the Universidade Católica Portuguesa (FMD-UCP, Viseu, Portugal), in the school year 2021–2022. This study focused on the latest confinement periods for the year 2022. The data collected were used exclusively for this study. After its completion and objectives, this data was eliminated, in conjunction with the questionnaire and its link to the Google Forms (Google Forms^®^; Google LLC, Mountain View, CA, USA) platform and the database in software Excel (Microsoft Excel v. 16.68, Microsoft Office, Redmond, WA, USA).

### 2.2. Questionnaires

Evaluation of DTM symptoms, oral behaviors, anxiety, and depression was performed using validated questionnaires already used in another similar study [18]. The questionnaire [35] was shared and applied during the period of May to June 2022. An email was sent to the students with the questionnaire access link. The three questionaries sent were:

A. Anxiety and Depression Questionnaire adapted from The Hospital of Anxiety and Depression Scale (HADS). HADS was used to evaluating the symptoms of participants’ anxiety and depression. Consisting of 14 questions, 7 evaluate anxiety (HADS-A) and the remaining 7 evaluate depression (HADS-D) [36]. Each item can be punctuated from 0 to 3, resulting in 21 points as the maximum for each scale.
B. DTM symptom questionnaire according to diagnostic criteria for temporomandibular dysfunctions. According to the Diagnostic Criteria for Temporomandibular Disorders: Clinical Protocol and Assessment Instruments (DC/TMD) Questionnaire. The participant was given the option to complete the questionnaire because the questions in it have an objective character about the symptoms of TMDs [18,37].
C. Questionnaire on the presence of habits for functional and oral behaviors, according to the DC/TMD Oral Behavior Checklist (OBC) questionnaire, an evaluation list of oral behaviors (ELOB). This questionnaire was also translated and validated in Portuguese. Participants answered objective questions about the frequency with which they carried out certain habits during sleep and while awake during the period in question. Five response options varied, and each had a given quotation, from 0 to 4, both at the time the participant was awake and when he was sleeping, and the options differed from each moment. Each participant was given a score that resulted from the sum of the quotations of the chosen answers. The greater the score, the greater the presence of oral behaviors [18].

### 2.3. Eligibility Criteria and Variables Studied

The inclusion considered those who were (i) students of Dental Medicine at FMD-UCP, and (ii) attending the third, fourth, and fifth years. For exclusion were those who (i) refused to participate or were unable to participate in the study, and (ii) were students of other courses.

The variables under study were (a) general information data, such as age, gender, marital status, level of schooling, the academic degree, the possible medication, and any existing pathologies; (b) questions related to taste changes or thoughts that participants may have identified, indicating (or not) worsening psychological conditions, such as anxiety and depression. Questions like, “I tasted similarly as before?”, “Did you laugh and have fun when you saw funny things?” and “Was it exciting when I thought about the future?” are examples of questions of this nature present in the questionnaire; (c) more technical questions about headaches, sounds and mandibular blocks that may have happened; (d) issues that shed light on how often the participant practiced some habits, first during sleep and then during the day, for example, the regularity with which the participant grinded or ranged their teeth, chewed elastic tablet, sang, yawned, or spoke for long periods, among others.

### 2.4. Positive Hypothesis Formulated

For the objectives of this study, the following positive hypotheses of the investigation were defined:

**H1.**  *There is a significant relationship between TMD and gender/age.*

**H2.**  *The frequency of oral behaviors is related to the existence of TMD.*

**H3.**  *The existence of TMD is related to the existence of anxiety and depression.*

**H4.**  *The academic degree contributes significantly to the existence of DTM, anxiety, and depression.*

**H5.**  *There is a significant relationship between the existence of anxiety and gender.*

**H6.**  *There is a significant relationship between the existence of depression and gender.*

**H7.**  *The existence of anxiety is significantly associated with age.*

**H8.**  *The existence of depression is significantly associated with age.*

**H9.**  *Students’ anxiety and depression contribute to the greater frequency of oral behaviors.*

### 2.5. Statistical Analysis

After collecting data directly from Google Forms (Google Forms^®^; Google LLC, Mountain View, CA, USA), they were transferred to an Excel (Microsoft Excel v. 16.68, Redmond, WA, USA) and then inserted into the IBM SPSS^®^ program (IBM Corp. Released 2019. IBM SPSS Statistics, Version 26.0. Armonk, NY, USA: IBM Corp) to be analyzed and interpreted. Descriptive analysis showed the distribution of absolute and relative frequencies, central tendency measures (average and median), and dispersion measures (standard deviation). Inferential statistical analysis was performed to verify the investigation’s hypotheses. A chi-square test allowed the independence between ordinal or nominal variables to be tested. The Spearman correlation test was also used to analyze the relationship between existent oral behaviors (OB) versus TMD, anxiety, and depression. All tests were done with a 95% significance, where results were considered significant if *p* ≤ 0.05.

## 3. Results

### 3.1. Sociodemographic Characterization

This study invited 170 students but enrolled 167 who answered the questionnaire (response rate of 98.2%). Of 167 students, most were female (*n* = 100, 59.9%) and never married (*n* = 145, 86.8%). The age group ranged from 19 to 53, averaging 23.5 (±6.7) years old. Students were divided into two age groups: one under 26 and the other at 26 or older (*n* = 138, 82.6%). The sample of third-year students was made up of 51 students (30.5%), and from the fourth and fifth years, 115 people (68.9%) (Table 1).

### 3.2. Descriptive and Statistical Analysis

#### 3.2.1. TMD

After analyzing the results, essential values in our study were found. Of the students who answered that they had headaches in the first question (11.4%), 4 (22.2%) reported that the pain had existed for 12 months, and 2 (11.1%) reported that the pain had existed for 24 months. Also, there was relevance in the question of joint sounds, since 22.2% (*n* = 37) had a positive response (Table 2). The results relating to TMD showed that the majority of students surveyed did not have this problem (*n* = 109, 65.3%), while 19 students (11.4%) had a positive response for pain and 39 students (23.4%) had TMD but without pain.

#### 3.2.2. Anxiety and Depression (HADS)

As for the HADS questionnaire, only the responses of 165 students (99.98%) were counted, since the other two (0.012%) were, during the period in question, taking medication for anxiety or depression. Questions such as “I felt tense or constricted”, “I felt a kind of fear as if something negative was going to happen”, “I had my head full of worries”, “I felt happy”, “Was I able to sit at the I felt at ease and relaxed”, “I was slow to think and do things”, “I had a feeling of fear like butterflies or tightness”, “I lost interest in taking care of my appearance”, and “I felt excited when thinking about the future” recorded the highest percentage of answers in options with a positive value for the scale in question, that is, in these questions, most people showed signs, some greater and others less, of suffering from anxiety and depression.

The question “My head was full of worries”, the question related to anxiety, and “I lost interest in taking care of my appearance”, the question related to depression, which is also mentioned above, saw 9.0% and 8.4% of participants (*n* = 15 and *n* = 14, respectively), choosing the option with a score of 3, in this case, “Most of the time” and “Completely” (Table 3).

Analyzing the HADS results, it was verified, considering the mean values obtained, that participants’ levels of anxiety (5.73 ± 3.82) and depression (5.36 ± 3.13) were both reduced. More specifically, considering a cutoff point established by the value of eight points, we noted that only 29.09% (*n* = 48) had anxiety symptoms (Table 4).

Regarding depression, also considering a cutoff in eight points, most participants were not suffering from depression (*n* = 126, 75.5%), and only 24.2% (*n* = 40) had some symptoms.

#### 3.2.3. Oral Behavior (OB)

Regarding the frequency of OB, all behaviors have the answer “None” as a majority, except for “Yawn”, where there were 68 people (40.7%) who answered, “A little part of the time” (Table 5). Only a small percentage of students chose the “All the time” option in any of the 19 options. More specifically, according to the average and median values obtained, this tendency for students to “yawn” a small part of the time and “eat between meals” was noted. As for the OBs performed during sleep, both by the frequency of cases and by the measures of central tendency (average and median) it was shown that there was not a propensity for students to have OBs during sleep, such as clenching or grinding their teeth or, less than one night a month, sleeping in a position that puts pressure on the jaw, even though 24% (*n* = 40) of participants responded that they slept between four and seven nights a month (Table 6). Lastly, we noticed that the general results of the OBC scale were also reduced according to the mean values obtained (11.87 ± 10.24) in an interval that varied between a minimum of zero and a maximum of 52 points (Table 4).

#### 3.2.4. Correlation between TMD and Gender, Age, Academic Degree, Anxiety, and Depression

From the correlation results (Table 7), there was a statistically significant association between the existence of TMD and gender (x^2^ = 8.47, *p* = 0.014), with more cases within the female gender between TMD and anxiety (x^2^ = 29.52, *p* < 0.001), with more TMD cases in students with anxiety (62.5%) compared to those without anxiety (23.1%), and between TMD and depression (x^2^ = 13.07, *p* = 0.001). Within the group of students without anxiety but with TMD, there was a significant difference in percentage for those who had pain or not, with the last having a higher proportion. For the group with anxiety and TMD, those with pain and without pain presented very similar proportions. Also, there was a higher proportion of patients in the group with TMD and pain for participants with depression (22.5%) compared to those without depression (8.0%). In the group with TMD without pain, there was also a higher percentage of participants with depression (35.0%).

There was no statistically significant association between TMD and students’ age (x^2^ = 4.96, *p* = 0.084). However, it was possible to verify that the proportion of TMD cases was higher in participants younger than 26 (38.4%) and, in both age groups, the percentage of TMD with pain was higher. A similar result was found between TMD and academic degree (x^2^ = 8.34, *p* = 0.080), with no statistically significant association, differing only for pain.

For the analysis of anxiety (Table 8), there was a statistically significant association of anxiety with gender (x^2^ = 12.74, *p* = 0.0001). The proportion of women (39.4%) was higher in the anxious students’ group than in males (13.6%). On the other hand, there was no association between anxiety and age (x^2^ = 0.16, *p* = 0.692) or anxiety and academic degree (x^2^ = 0.53, *p* = 0.767). With the values collected, almost 75% of students in the older age group did not have anxiety, whereas in the younger group the value was around 70%. Moreover, in the group of students without anxiety, the highest percentage belonged to the group that only completed the high school level. On the contrary, the highest percentage of students with anxiety belonged to the master’s or doctorate group.

Regarding the association between depression and gender, there was a statistically significant result (x^2^ = 4.95, *p* = 0.026), with a higher proportion of women (30.3%) affected. Between age and depression, no statistically significant result was found (x^2^ = 3.03, *p* = 0.082). However, it should be noted that the older age group had a lower percentage of cases of depression (11.1%). On the other hand, in the younger group, the opposite was observed, with a higher percentage of cases of this psychological disorder (26.8%). Like the anxiety result, academic degree was also not significantly associated with depression (x^2^ = 1.96, *p* = 0.375). In this study, for students without depression, the highest percentage belonged to the group that had a degree as an academic degree. On the contrary, in the group of students with depression, the highest percentage was in the group with a master’s or doctoral degree (Table 8).

#### 3.2.5. Association between OBs and Anxiety, Depression, and TMD

In the cases involving OBs, Spearman’s correlation was used to analyze the data collected with data from anxiety, depression, and TMD. Table 9 presents the results of those correlations, which were statistically significant for anxiety (rs = 0.36, *p* = 0.0001), depression (rs = 0.22, *p* = 0.006), and TDM (rs = 0.39, *p* = 0.0001). Through those data, it was confirmed that there are correlations between OB and the three variables verified, with positive coefficient values for all, represented by an increase in one of the variables, which will also increase the others (Table 9).

## 4. Discussion

The goal of this study was to describe and compare the possible impact of compulsory confinement periods related to SARS-CoV2 on 167 Portuguese students’ health. A similar study was developed in Brazil in 2020 [18], which enrolled 147 students. Among the participants, the mean age was 23.5 (±6.7 years) and there was a predominance of females (59.9%). Data similar were observed in the literature, with a high presence of women, such as 54.7% [2], 77% [18], and 80.3% [38]. Also, similar ages were reported, with mean ages of 21.76 years (±1.859) [2] and 21.46 years (±2.37) [18].

Following Medeiros et al. [18], TMD was classified in our study into the following three classes: (i) the existence of TMD symptoms (headaches) with pain, (ii) the existence of TMD symptoms without pain (without headaches but with other symptoms [jaw locks or joint sounds]), and (iii) the absence of TMD symptoms (without headaches or other types of pain). The prevalence of TMD here was 35%. Although there are no studies in Portugal analyzing this prevalence in the COVID-19 period, we were able to compare these results with another international study [18], which had values slightly higher (54.8%, *n* = 62); however, 50% (*n* = 31) of participants reported having pain and the other 50% not. In another study by Sójka et al. [39] in Poland, where a questionnaire was also presented, 33.2% of the participants had TMD symptoms. This study revealed data more similar to those of our investigation.

The presence of anxiety and depression was found in, respectively, 24.2% and 29.1% of the participants. Although we did not find any studies that calculated the prevalence of anxiety and depression in Portuguese Dentistry students, it seems plausible based on the existing literature. There are studies [2,40,41,42] that show a higher prevalence of anxiety and depression than those obtained in this investigation. More specifically, a study conducted in Brazil during the pandemic [18] showed higher percentages of depression and anxiety (49.6% and 38.9%, respectively). It is important to note that these high levels of anxiety and depression can also be attributed to the stress experienced by dental students during the academic period [2,18,43,44,45,46]. In a study evaluating dental professionals (dentists and dental hygienists) from Italy, 30% of dentists and 27% of hygienists declared that they suffered from work-related stresses during the pandemic [47].

The relationship between the student’s gender and TMD indicated a statistically significant association (*p* = 0.014), suggesting a higher prevalence in the female gender. However, these results are not in line with the previous study [18] carried out in 2020, also during the pandemic period, which stated that there were no differences between genders regarding the existence of TMD. The literature reported that the prevalence of TMD in females is almost twice as high as in males (Sanders et al. [48], Huang et al. [49], and Graue et al. [50]. According to Magnusson et al. [51] and Pedroni et al. [52], this may be justified by differing physiological characteristics, such as hormonal variations, different characteristics of connective tissues, and muscle structure. Moreover, a meta-analysis before the pandemic by Bueno et al. [53] reported a higher prevalence and greater tendency for females to have TMD than males. In 2021, another study [54] carried out in South Korea revealed more TMD symptoms in females, who presented a higher prevalence of pain and muscle palpitations compared to males.

Another objective defined by the present study was to analyze the relationship between TMD symptoms and students’ anxiety and depression. The results obtained point to a statistically significant association between TMD and anxiety (*p* < 0.001) and depression (*p* = 0.001). There was a positive and direct correlation: students presenting TMD symptoms were more likely to reveal anxiety and depression or, at least, were more likely to suffer from these conditions. These results are in line with previous studies by Kindler et al. [55], Vedolin et al. [44], and List et al. [56] that confirm this association. The possible explanations for these relationships stem from the fact that those symptoms can trigger muscle hyperactivity, followed by dystrophy and mechanical alteration of the muscles, eventually causing muscle pain [55,57]. In addition, they can stimulate joint inflammation with subsequent biomechanical changes, causing joint pain [55,57]. All these factors, combined with stress and anxiety, increase the activity of the sympathetic system, as well as the release of epinephrine, which can potentiate the action of acetylcholine, trigging a cascade of events that will end with a lowered pain threshold in muscle nociceptors [44,58,59,60,61]. Otherwise, Medeiros et al. [18] did not find a significant association between these variables.

There were statistically significant relationships between the student’s gender and their levels of anxiety and depression, respectively, (*p* = 0.0001) and (*p* = 0.026), with females tending to be more anxious and depressed. This fact has also been observed in previous studies conducted during the COVID-19 pandemic. Hakami et al. [2], Silva et al. [38], and Özdin et al. [62] found these same differences. In contrast, Wang et al. [63] during the pandemic and Asher et al. [64] in 2017 (pre-pandemic) found a difference only for anxiety, noting that this disorder was threefold more frequent in females than in males. It should be noted that these differences between genders were already observed pre-pandemic, according to the results of previous investigations in Saudi Arabia [42] and Pakistan [65]. A possible explanation may involve the fact that females are more concerned about their health and also because of the increase in cases of domestic violence during the pandemic [38,66].

In our study, we evaluated the relationship between anxiety and depression at the academic level, and it was concluded that none of them were statistically significant (*p* = 0.767 and *p* = 0.375, respectively). Even so, it was possible to observe that both conditions were more prevalent in students with a higher academic degree. Studies in the literature corroborate these data [46,67]. Although in quite different periods, Naidu et al. [46] in 2002 and Shamsuddin et al. [68] in 2013 showed that students with a higher academic experience had more stress and psychological disturbances than students with lower academic degrees.

For the relationship between OBs and levels of anxiety and depression, there were statistically significant results (*p* = 0.0001 and *p* = 0.006, respectively). Thus, these data confirm the conclusions of Chow et al. [68] and Machado et al. [45], which refer to a direct bidirectional relation between the frequency of OBs and anxiety, especially if pain is present. This statistically significant association of anxiety and depression in our study with the existence of OBs was already found during the COVID-19 pandemic by Medeiros et al. [18].

Therefore, the relationship between OBs and TMD was significant (*p* = 0.0001), confirming the statements of previous authors [68]. They stated that some habits, such as “clenching the teeth”, “leaving the jaw in a rigid position”, “pressing the tongue against the teeth”, and “moving the tongue, mouth, and cheeks a lot”, were associated with the existence of TMD with pain. This fact can be explained due to the necessity of mandibular muscle contraction, which can lead to excessive effort and, subsequently, ischemia and pain in the region [69,70]. These results agree with results obtained in a more recent study [18], which showed that the existence of TMD contributed to the performance of OBs.

### Study Limitations and Prospects

The questionnaire was carried out by the participants during May and June of the year 2022, based on activities lived and experienced in January. Therefore, it is presumable that there was occurrence of bias in the answers due to the interval. It must be considered that the diagnosis of TMD was based on clinical components, with the need for intra and extraoral assessment. In this study, only a questionnaire was used, and positive symptoms of this condition were reported only with answers to the questions, which may represent a limitation.

It would be useful to repeat the application of the questionnaires to the research participants to investigate the occurrence of signs and symptoms of TMD that may have evolved into post-traumatic stress syndrome [18,71]. In addition, since few studies are exploring these relationships in the university environment, carrying out the same research with a greater sample size and multicenter is recommended.

Moreover, it is suggested to revise the content to take into account new procedures involving the personal protective equipment (PPE) that is used on a daily basis by students and professionals, as well as the other necessary cares taken in the office to avoid contamination and the propagation of diseases. For the period after COVID-19, multidisciplinary involvement for the students, such as with psychologists, psychiatrists, physicians, and dentists, is necessary in order to observe and treat the students due to the possible traumatic period lived.

## 5. Conclusions

According to the objectives proposed, it was possible to conclude there was a 35% prevalence in TMD, 29% in anxiety, and 24% in depression, with statistically significant relationships between TMD, anxiety, and depression and gender and frequency of OBs. Otherwise, no significant association was found for TMD, anxiety, and depression with students’ age and academic degree. Also, greater TMD and anxiety corresponded to greater OBs, and a direct relation was observed for OBs and depression and TMD.

## Figures and Tables

**Table 1:** Student sample characterization.

		*n*	%
Gender	Female	100	59.9
	Male	67	40.1
Age		Average (±SD)	Minimum/Maximum
		23.5 (6.7)	19/53
Groups by age	<26	138	82.6
	≥26	29	17.4
Marital status	Married	12	7.2
	Never married	145	86.8
	Separated	6	3.6
	Union	3	1.8
	Widower	1	0.6
Academic level	High school	56	33.5
	College	95	56.9
	Master or PhD	16	9.6
Currently studying	Dental Medicine—3rd year	51	30.5
	Dental Medicine—4th and 5th years	115	68.9
	Higher levels	1	0.6

**Table 2:** Frequency of responses to TMD items.

Temporomandibular Disorders (TMD) Questions		*n*	%
In the mentioned period, did you have some headaches that included the sources of your head? *	No	148	88.6
	Yes	19	11.4
How many months ago your headache arose in the source zone for the first time?	1	3	16.7
	2	2	11.1
	4	1	5.6
	6	3	16.7
	7	1	5.6
	12	4	22.2
	24	2	11.1
	26	1	5.6
	30	1	5.6
In the mentioned period, did the following activities alter a headache in the source zone on any side?	No response	138	82.6
	No	22	13.2
Chew hard or hard food	Yes	7	4.2
In the mentioned period, did the following activities alter a headache in the source zone on any side? Open your mouth, or move your jaw forward or side	No response	138	82.6
	No	20	12.0
	Yes	9	5.4
In the period mentioned, did the following activities change any source-zone headaches on either side? Habits with the jaws, such as keeping the teeth together, clenching/grinding the teeth, or chewing gum	No response	138	82.6
	No	17	10.2
	Yes	12	7.2
In the period mentioned, did the following activities change any source-zone headaches on either side? Other jaw activities like talking, kissing, or yawning	No response	139	83.2
	No	24	14.4
	Yes	4	2.4
Did you have any joint sound (or sounds) when you moved or used your jaw during the mentioned period? *	Right	4	2.4
	Left	3	1.8
	No	110	65.9
	Do not know	13	7.8
	Yes	37	22.2
Have you ever had your jaw blocked or trapped, even for a moment, so you didn’t open it FULLY? *	Left	1	0.6
	No	144	86.2
	Do not know	5	3.0
	Yes	17	10.2
Is the jaw blocked or trapped severely enough to limit opening and interfere with your feeding ability?	No response	131	78.4
	No	30	18.0
	Do not know	1	0.6
	Yes	5	3.0
In the past 30 days, did your jaw lock so that you could not open it FULLY, even for a moment, and then unlock it to open it FULLY?	No response	130	77.8
	No	29	17.4
	Yes	8	4.8
Is your jaw blocked or limited, so it does not open FULLY?	No response	143	85.6
	No	23	13.8
	Do not know	1	0.6
In the last 30 days, when you opened your mouth a lot, did your jaw lock or catch even for a moment so that you couldn’t close it from this wide opening position? *	Right	1	0.6
	No	157	94.0
	Do not know	2	1.2
	Yes	7	4.2
In the last 30 days, when your jaw was locking or binding with your mouth wide open, did you have to do anything to get it closed, including resting, moving, pushing, or maneuvering it?	No response	138	82.6
	No	23	13.8
	Do not know	2	1.2
	Yes	4	2.4

* Compulsory questions.

**Table 3:** Frequency of responses to HADS items.

Hads	0		1		2		3		NR	
	*n*	%	*n*	%	*n*	%	*n*	%	*n*	%
I felt tense or constricted	45	26.9	87	52.1	29	17.4	4	2.4	2	1.2
I had a taste for the same things as before	90	53.9	64	38.3	8	4.8	3	1.8	2	1.2
I felt a kind of fear, as if something negative was going to happen	56	33.5	62	37.1	39	23.4	8	4.8	2	1.2
I laughed and had fun when I saw funny things	116	69.5	43	25.7	6	3.6	0	0	2	1.2
My head was full of worries	48	28.7	72	43.1	30	18	15	9	2	1.2
I felt happy	38	22.8	98	58.7	27	16.2	2	1.2	2	1.2
I was able to sit at ease and feel relaxed	60	35.9	72	43.1	33	19.8	0	0	2	1.2
I was slow to think and do things	38	22.8	85	50.9	34	20.4	8	4.8	2	1.2
I had a feeling of fear, like butterflies in his stomach or a tightness	77	46.1	77	46.1	11	6.6	0	0	2	1.2
I lost interest in taking care of my appearance	54	32.3	72	43.1	25	15	14	8.4	2	1.2
I felt restless, as if I could not sit still	72	43.1	65	38.9	21	12.6	7	4.2	2	1.2
I felt excited when thinking about the future	67	40.1	69	41.3	25	15	4	2.4	2	1.2
I suddenly had a feeling of panic	109	65.3	37	22.2	15	9	4	2.4	2	1.2
I was able to feel pleasure when watching a good program on television or radio or when I read something	80	47.9	64	38.3	18	10.8	3	1.8	2	1.2

**Table 4:** Frequency of anxiety, depression, and OBs in the sample distribution.

Variable	Average (± SD)	Minimum–Maximum
HADS—Anxiety	5.73 (±3.82)	(0–17)
HADS—Depression	5.36 (±3.13)	(0–13)
OBs	11.87 (±10.24)	(0–52)

**Table 5:** Results related to OBs questions during the day.

	Not Once		A Little Part of the Time		Some Part of the Time		Most of the Time		All the Time		Average	Median
I gnashed his teeth during his waking hours	129	77.2%	26	15.6%	8	4.8%	3	1.8%	1	0.6%	0.33	0
Clenched teeth during waking hours	98	58.7%	38	22.8%	24	14.4%	5	3%	2	1.2%	0.65	0
Pressed, touched, or held teeth together other than to eat (i.e., contact between upper and lower teeth)	79	47.3%	46	27.5%	29	17.4%	9	5.4%	4	2.4%	0.88	1.0
Grasping, squeezing, or creating muscle tension without grinding or bringing teeth together	102	61.1%	34	20.4%	23	13.8%	7	4.2%	1	0.6%	0.63	0
Maintained or projected the mandible forward or to the side	127	76%	24	14.4%	14	8.4%	1	0.6%	1	0.6%	0.35	0
I pressed his tongue hard against his teeth.	124	74.3%	28	16.8%	11	6.6%	2	1.2%	2	1.2%	0.38	0
Put your tongue between your teeth	126	75.4%	21	12.6%	15	9%	3	1.8%	2	1.2%	0.41	0
Biting, chewing, or playing with your tongue, cheeks, or lips	103	61.7%	36	21.6%	21	12.6%	5	3%	2	1.2%	0.60	0
I held the jaw in a rigid or tense position as if preparing for an impact or protecting the jaw.	127	76%	24	14.4%	11	6.6%	5	3%	0	0	0.37	1.0
Kept between teeth or bit objects, such as hair, pipe, pencils, pens, fingers, nails, etc.	108	64.7%	30	18%	20	12%	6	3.6%	3	1.8%	0.6	0
Chewing gum	77	46.1%	46	27.5%	27	16.2%	12	7.2%	5	3%	0.93	1.0
Played a musical instrument that involved the use of the mouth or jaw (for example, brass, woodwind, or string instruments)	151	90.4%	7	4.2%	8	4.8%	1	0.6%	0	0	0.16	0
Leaning with the jaw over hand, e.g., cupping or resting chin on hand	85	50.9%	38	22.8%	31	18.6%	10	6%	3	1.8%	0.85	0
Chewing food on one side	107	64.1%	37	22.2%	21	12.6%	2	1.2%	0	0	0.51	0
Eat between meals (i.e., food that requires chewing)	64	38.3%	42	25.1%	35	21%	17	10.2%	9	5.4%	1.19	1.0
Talked for extended periods (e.g., taught, sold, customer support)	121	72.5%	27	16.2%	12	7.2%	6	3.6%	1	0.6%	0.44	0
Sang	89	53.3%	46	27.5%	20	12%	7	4.2%	5	3%	0.76	0
Yawned	45	26.9%	68	40.7%	33	19.8%	14	8.4%	7	4.2%	1.22	1.0
I held the phone between his head and shoulders	101	60.5%	40	24%	19	11.4%	5	3%	2	1.2%	0.6	0

**Table 6:** Results for questions related to OBs during sleep.

	None		Less than 1 Night/Month		1–3 Nights/Month		1–3 Nights/Week		4–7 Nights/Month		Average	Median
Clenched or ground teeth in sleep, based on any information he/she might have	94	56.3%	28	16.8%	16	9.6%	18	10.8%	11	0.6%	0.95	0
Sleeping in a position that puts pressure on the jaw (e.g., on your stomach, on your side)	66	39.5%	18	10.8%	22	13.2%	21	12.6%	40	24%	1.71	1.0

**Table 7:** Association between TMD and depression/anxiety/academic degree/age/gender.

	Without Depression (<8), *n* (%)	With Depression (≥8), *n* (%)	x^2^ (*p*)	Without Anxiety (<8), *n* (%)	With Anxiety (≥8), *n* (%)	x^2^ (*p*)	High School *n* (%)	College *n* (%)	Master/Ph.D. *n* (%)	x^2^ (*p*)	<26 y. *n* (%)	≥26 y. *n* (%)	x^2^ (*p*)	Female *n* (%)	Male *n* (%)	x^2^ (*p*)
without TMD	91 (72.8%)	17 (43.5%)	13.07 (0.001)	90 (76.9%)	18 (37.5%)	29.52 (<0.001)	36 (64.3%)	61 (64.2%)	12 (75%)	8.34 (0.08)	85 (61.6%)	24 (82.8%)	4.96 (0.084)	59 (59%)	50 (74.6%)	8.47 (0.014)
TMD with pain	17 (43.5%)	9 (22.5%)		5 (4.3%)	14 (29.2%)		17 (30.4%)	18 (18.9%)	4 (25%)		35 (25.4%)	4 (13.8%)		17 (17%)	2 (3%)	
TMD without pain	25 (19.2%)	14 (35%)		22 (18.8%)	16 (33.3%)		3 (5.4%)	16 (16.8%)	0 (0%)		18 (13%)	1 (3.4%)		24 (24%)	15 (22.4%)	
Total	125 (100%)	40 (100%)		117 (100%)	48 (100%)		56 (100%)	95 (100%)	16 (100%)		138 (100%)	29 (100%)		100 (100%)	67 (100%)	

**Table 8:** Association between anxiety and depression and academic degree, age, and gender.

	High School *n* (%)	College *n* (%)	Master/Ph.D. *n* (%)	x^2^ (*p*)	<26 y.o. *n* (%)	≥26 y.o. *n* (%)	x^2^ (*p*)	Female *n* (%)	Male *n* (%)	x^2^ (*p*)
without anxiety (<8)	41 (74.5%)	65 (69.1%)	11 (68.8%)	0.53 (0.767)	97 (70.3%)	20 (74.1%)	0.16 (0.692)	60 (60.6%)	57 (86.4%)	12.74 (0.00001)
with anxiety (≥8)	14 (25.5%)	29 (30.9%)	5 (31.3%)		41 (29.7%)	7 (25.95%)		39 (39.4%)	9 (13.6%)	
Total	55 (100%)	95 (100%)	16 (100%)		138 (100%)	27 (100%)		99 (100%)	66 (100%)	
without depression (<8)	39 (70.9%)	75 (79.8%)	11 (68.8%)	1.96 (0.375)	101 (73.2%)	24 (88.9%)	3.03 (0.082)	69 (69%)	56 (84.8%)	4.95 (0.026)
with depression (≥8)	16 (29.1%)	19 (20.2%)	5 (31.3%)		37 (26.8%)	3 (11.1%)		30 (30.3%)	10 (15.2%)	
Total	55 (100%)	94 (100%)	16 (100%)		138 (100%)	27 (100%)		99 (100%)	66 (100%)	

**Table 9:** Spearman correlation between OBs and TMD, anxiety, and depression.

OBC	DTM	Anxiety	Depression
Correlation coeficient	0.391	0.361	0.215
*p*	0.0001	0.0001	0.006
N	167	165	165
